# Relationship between perceived physical literacy and obesity-related outcomes in adolescents: the EHDLA study

**DOI:** 10.3389/fpubh.2024.1321361

**Published:** 2024-04-16

**Authors:** Gabriel Domínguez-Martín, Pedro J. Tárraga-López, José Francisco López-Gil

**Affiliations:** ^1^Consejería de Educación, Region of Murcia, Murcia, Spain; ^2^Departamento de Ciencias Médicas, Facultad de Medicina, Universidad de Castilla-La Mancha, Albacete, Spain; ^3^One Health Research Group, Universidad de Las Américas, Quito, Ecuador

**Keywords:** physical education, physical fitness, adiposity, anthropometric indicators, body mass index, waist circumference, overweight, youths

## Abstract

**Purpose:**

The aim of the present study was to examine the relationship between perceived physical literacy and obesity-related outcomes among adolescents from Spain.

**Methods:**

This is a secondary cross-sectional analysis including a total sample of 845 Spanish adolescents (55.3% girls) aged 12–17 years from the *Valle de Ricote* (Region of Murcia) from the Eating Healthy and Daily Life Activities (EHDLA) project. Physical literacy was evaluated using the Spanish Perceived Physical Literacy Instrument for adolescents (S-PPLI). Body mass index was computed by taking the participants’ body weight in kilograms and dividing it by the square of their height in meters, and body mass index (*z* score) and overweight/obesity and obesity were computed by the World Health Organization age- and sex-specific thresholds. Waist circumference was measured using a constant tension tape. Moreover, the waist-to-height ratio was calculated, and therefore, abdominal obesity was determined. Skinfold measurements were taken at the triceps and medial calf using calibrated steel calipers.

**Results:**

In general, the overall trend was downward (i.e., the higher the PPLI score the lower the obesity-related indicators), with the approximate significance of smooth terms being statistically significant for all models examined (*p* < 0.001). Adolescents with lower perceived physical literacy (PPL) showed the highest estimated marginal means of body mass index, body mass index *z* score, waist circumference, waist-to-height ratio, and skinfold (triceps and calf) and predictive probabilities of having excess weight, obesity, and abdominal obesity, while their counterparts with high PPL had the lowest. In addition, significant differences were observed for all the obesity-related indications between adolescents with low PPL and those with medium PPL (*p*-adjusted < 0.05 for all indicators), as well as with those with high PPL (*p*-adjusted < 0.05 for all indicators). Moreover, these significant differences were also shown for most indicators between adolescents with medium PPL and those with high PPL (except for obesity).

**Conclusion:**

Physical literacy could play a crucial role in maintaining more desirable obesity-related outcomes in adolescents. Adolescents with high perceived physical literacy showed lower obesity-related indicators (i.e., body mass index, body mass index *z* score, waist circumference, waist-to-height ratio, skinfolds), as well as a lower probability of having excess weight, obesity, and abdominal obesity.

## 1 Introduction

Obesity during adolescence has been linked with adverse health outcomes that include type 2 diabetes, dyslipidemia, non-alcoholic fatty liver disease, mental health disorders and social stigma (among others) (1). This fact has led to its conception as a severe public health problem of the 21st century (2). In 2019, the World Obesity Federation predicted that by 2025, there would be approximately 206 million children and teenagers aged 5–19 with obesity, and this number would rise to approximately 254 million by 2030 (3). The most recent Physical Activity, Sedentarism, Lifestyles, and Obesity in Spanish Youth (PASOS) study (4), conducted in Spain, revealed that in 2022–2023, the rates of overweight, obesity, and excess weight were 21.6, 11.6, and 33.2%, respectively, based on the criteria set by the World Health Organization (WHO) (5). In spite of these alarming statistics, the WHO has recently cautioned that no European nation is making adequate strides toward mitigating the growing problem of overweight and obesity by the year 2025 (6).

Numerous studies indicate that promoting and sustaining healthy lifestyle behaviors can serve as an effective approach to prevent obesity among the young population (7–10). In recent times, there has been a significant surge in the attention given to physical literacy, leading to the emergence of various programs, curricula, and policies aimed at enhancing it (11, 12). This seems to be logical considering the beneficial associations observed between active lifestyles and various health indicators (13, 14). Physical literacy involves developing the desire, confidence, skills, knowledge, and understanding necessary for lifelong physical activity (15, 16). It is cultivated through active participation in physical pursuits (17) and represents a distinct form of intelligence that extends beyond mere exertion, serving as a vital foundation for sustained engagement in physical activities (18).

Initially, it was theorized that physical literacy should be related to physical activity, physical fitness, motor competence, and weight status (16). Motor illiteracy encompasses challenges in motor development, including declines in competence and confidence, particularly exacerbated by the existence of adverse conditions like obesity, which serves as a tangible obstacle (19, 20). However, to date, only a few studies have studied the association of physical literacy with obesity-related outcomes (21, 22). For instance, one study among French adolescents found an inverse association between physical literacy and body fat percentage (22). This same inverse relationship was observed in another study among Canadian children (21). Due to the limited research on this specific association, a better understanding of how physical literacy is linked with obesity-related outcomes might be relevant in developing formulating future intervention initiatives aimed at preventing or reducing obesity among adolescents. Thus, the aim of the present study was to examine the relationship between perceived physical literacy (PPL) and obesity-related outcomes among adolescents from Spain.

## 2 Materials and methods

### 2.1 Study design and population

The present cross-sectional study is a secondary analysis with data from the Eating Healthy and Daily Life Activities (EHDLA) project. The EHDLA study protocol has been published elsewhere (23). The adolescents involved in this research were Spanish students (aged 12–17 years) attending the three secondary schools in the *Valle de Ricote* in the Region of Murcia. Data were gathered during the 2021–2022 academic year. Of the initial 1,378 adolescents (100.0%) from the EDHLA study, 118 (8.6%) were eliminated from the study due to lack of anthropometric information. Moreover, additional participants were eliminated due to lack of data regarding PPL (*n* = 239; 17.3%) and energy intake (*n* = 176; 12.8%). Thus, 845 adolescents (55.3% girls) were included in this cross-sectional study. Written consent was obtained from parents or guardians of the teenage participants in this study. The participants were provided with detailed information about the study’s objectives, as well as the assessments and surveys that would be conducted. In addition, the adolescents themselves were asked to give their consent to participate.

This study was approved by the Bioethics Committee at the University of Murcia (Approval ID: 2218/2018), the Ethics Committee of the Albacete University Hospital Complex, and the Albacete Integrated Care Management (Approval ID: 2021–85). The research was conducted in accordance with the principles outlined in the Helsinki Declaration.

### 2.2 Procedures

#### 2.2.1 Perceived physical literacy (independent variable)

The Spanish Perceived Physical Literacy Instrument for Adolescents (S-PPLI) (24) was used to assess PPL in this study. The S-PPLI has been previously validated for use with Spanish youth. The original Perceived Physical Literacy Instrument (PPLI) was developed for physical education teachers and consisted of 18 items (25). However, the version used for adolescents in this study included 9 items. Participants rated these items on a 5-point Likert scale, ranging from 1 (strongly disagree) to 5 (strongly agree). The 9 items of the S-PPLI were evenly distributed across three categories: knowledge and comprehension, self-expression and interaction with others, and self-perception and self-confidence.

#### 2.2.2 Obesity-related indicators (dependent variables)

The body weight of the adolescents was measured using an electronic scale (with an accuracy of 0.1 kg) (Tanita BC-545, Tokyo, Japan), while height was determined by a portable height rod with an accuracy of 0.1 cm (Leicester Tanita HR 001, Tokyo, Japan). Body mass index (BMI) was computed by taking the participants’ body weight in kilograms and dividing it by the square of their height in meters. Furthermore, the BMI z score was computed by the World Health Organization (WHO) age-specific and sex-specific thresholds (26). Subsequently, the BMI *z* scores obtained were used to determine excess weight (i.e., overweight and obesity). Waist circumference was measured to the nearest 0.1 cm at the level of the umbilicus using a constant tension tape. Moreover, the waist-to-height ratio (WHtR) was calculated, and therefore, a value ≥0.5 was used as a cutoff point to determine abdominal obesity (27). Skinfold measurements were taken at the triceps and medial calf using calibrated steel calipers (Holtain, Crosswell, Crymych, United Kingdom). The measurements were taken to the nearest 0.2 mm. Both triceps and medial calf skinfolds have been shown to be effective in estimating body fat percentage in children and adolescents (28).

#### 2.2.3 Covariates

Adolescents provided self-reported information about their sex and age. The Family Affluence Scale (FAS-III) (29) was used to assess socioeconomic status. This involved summing up responses from six items related to their family’s possessions and amenities, such as bedrooms, vehicles, bathrooms, computers, travels, or dishwashers. The resulting FAS-III score ranged from 0 to 13, with higher scores indicating a higher socioeconomic status. Adherence to the Mediterranean Diet was evaluated using the Mediterranean Diet Quality Index in children and adolescents (KIDMED) (30). Energy consumption was assessed using a self-administered food frequency questionnaire that had been previously validated for use in the Spanish population (31). The overall sleep duration was determined by asking adolescents about their typical bedtime and wake-up time on both weekdays and weekends. The average sleep duration during weekdays and weekends was calculated using the formula [(average sleep duration on weekdays × 5) + (average sleep duration on weekends × 2)] divided by 7. To assess physical activity and sedentary behavior, the Youth Activity Profile Physical (YAP) questionnaire was used (32). This self-administered questionnaire covered a 7-day period and included 15 different items categorized into sections such as out-of-school activities, school-related activities, and sedentary habits.

### 2.3 Statistical analysis

Methods like density and quantile-quantile plots were employed to assess the normality of the variables, along with the utilization of the Shapiro–Wilk test. Descriptive data for categorical variables are presented as the number (*n*) and percentage (%) of observations in each category. For continuous variables, descriptive data are presented as the median and interquartile range of the values. The relationship between PPL status and obesity-related outcomes was examined using the chi-square test. As there was no interaction between sex and any obesity-related indicator (*p* > 0.05 for all), both boys and girls were analyzed together. To examine the association between the PPL score and obesity-related indicators among adolescents, generalized additive models (GAMs) were used. GAMs are flexible models that can capture non-linear relationships in the data without requiring a predefined mathematical structure. Restricted maximum likelihood (REML) was used for smoothness selection (33), and a shrinkage approach was employed with thin plate regression spline smoothers (34). The degree of non-linearity was quantified using the effective degrees of freedom (*edf*) of the GAM. Furthermore, generalized linear models (GLMs) were conducted to examine the relationship between PPL status (low, medium, high) and obesity-related indicators. For these analyses, we utilized a non-parametric bias-corrected and accelerated bootstrap method with 1,000 samples. Subsequently, a correction for multiple comparisons was applied using the false discovery rate *p*-value method proposed by Benjamini and Hochberg (35). For both GAMs and GLMs, estimated marginal means of BMI, BMI z score, WC, WHtR, and skinfold (triceps and calf) or predictive probabilities and their 95% confidence intervals (CIs) of having excess weight, obesity, and abdominal obesity were also estimated. All the models were adjusted for several covariates, including sex, age, socioeconomic status, adherence to the Mediterranean diet, energy intake, overall sleep duration, physical activity, and sedentary behavior. The statistical analyses were conducted using R statistical software (version 4.3.2) developed by the R Core Team in Vienna, Austria, and RStudio (2023.09.1 + 494) from Posit in Boston, MA, United States. Significance was determined at a threshold of *p* < 0.05.

## 3 Results

Table 1 presents the main characteristics of the adolescents examined. The highest proportion of adolescents with excess weight, obesity, and abdominal obesity was found in adolescents with low PPL (32.2, 12.3, and 28.1%, respectively). Conversely, the lowest proportion of these dichotomic obesity-related indicators was observed in adolescents with high PPL (excess weight: 18.2%; obesity; 6.1%; abdominal obesity: 12.8%).

**Table 1:** Main characteristics of the adolescents analyzed (*N* = 845).

Variables	Low PPL (9 to 31 points)	Medium PPL (32 to 36 points)	High PPL (37 to 45 points)
Participants (*n*, %)	292 (34.6)	240 (28.4)	313 (37.0)
Sex (girls, %)	186 (63.7)	124 (51.7)	157 (50.2)
Age (years)	14.0 (2.0)	14.0 (2.0)	13.9 (1.5)
FAS-III (score)	8.0 (3.0)	8.0 (2.2)	9.0 (3.0)
KIDMED (score)	6.0 (4.0)	7.0 (3.0)	8.0 (3.0)
Energy intake (kcal)	2635.3 (1499.5)	2577.7 (1500.9)	2555.7 (1463.8)
Overall sleep duration global (minutes)	492.9 (81.4)	497.1 (69.6)	497.1 (60.0)
YAP-S physical activity (score)	2.4 (0.9)	2.6 (0.8)	2.9 (0.9)
YAP-S sedentary behaviors (score)	2.6 (0.8)	2.6 (0.8)	2.4 (0.8)
BMI (kg/m^2^)	22.5 (7.1)	21.8 (5.9)	20.9 (5.4)
BMI (*z* score)	0.35 (2.10)	0.05 (1.99)	−0.27 (1.97)
Excess weight status (yes, %)	94 (32.2)	65 (27.1)	57 (18.2)
Obesity status (yes, %)	36 (12.3)	19 (7.9)	19 (6.1)
WC (cm)	72.0 (16.4)	71.4 (13.3)	69.3 (10.9)
WHtR [WC (cm)/height (cm)]	0.448 (0.097)	0.447 (0.078)	0.430 (0.061)
Abdominal obesity (yes, %)	82 (28.1)	49 (20.4)	40 (12.8)
Skinfold calf (mm)	17.0 (10.0)	15.0 (10.0)	14.0 (10.0)
Skinfold triceps (mm)	16.8 (10.0)	15.0 (10.0)	14.4 (9.0)

^*^Data reported as the median (interquartile range) or count (percentage). BMI, body mass index; FAS-III, Family Affluence Scale-III; KIDMED, Mediterranean Diet Quality Index in children and adolescents; MedDiet, Mediterranean, diet; Spanish Perceived Physical Literacy Instrument; YAP-S, Spanish Youth Active Profile; WC; waist circumference; WHtR, waist-to-height ratio.^a^According to the World Health Organization (26).^b^Using a cut-off point of waist-to-height ratio ≥ 0.5 (27).

**Figure 1:**
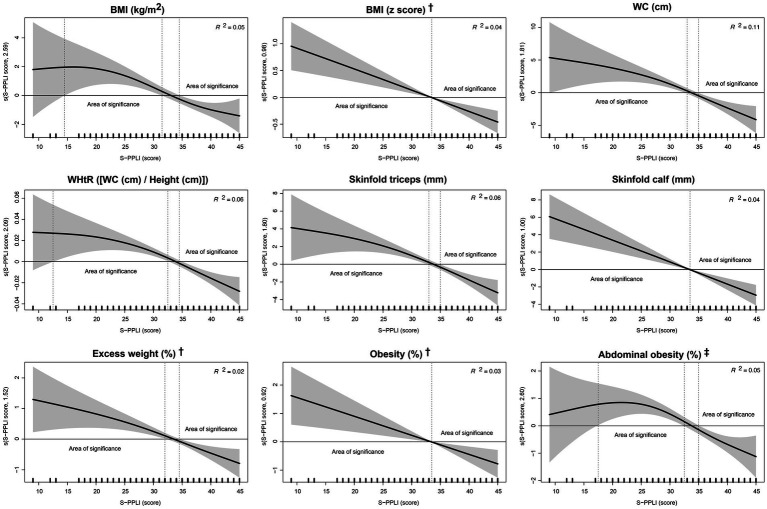
Association between perceived physical literacy score and obesity-related outcomes among adolescents using generalized additive models. BMI, body mass index; S-PPLI, Spanish Perceived Physical Literacy Instrument; WC, waist circumference; WHtR, waist-to-height ratio. Data expressed as standardized beta coefficient (black line) and 95% confidence interval (gray shadow). Adjusted for sex, age, socioeconomic status, adherence to the Mediterranean diet, energy intake, overall sleep duration, physical activity, and sedentary behavior. ^†^According to the World Health Organization (26). ^‡^Using a cut-off point of waist-to-height ratio ≥ 0.5 (27).

Figure 1 displays the GAMs for the relationship between the S-PPLI score and obesity-related indicators. In general, the overall trend was downward (i.e., the higher the PPLI score the lower the obesity-related indicators), with the approximate significance of smooth terms being statistically significant for all models examined (*p* < 0.001). Different significant areas were found in these associations. The first area indicated that the PPLI score was associated with BMI (from 14 to 31 points), BMI z score (from 9 to 33 points), WC (from 9 to 33 points), WHtR (from 13 to 32), skinfold (triceps) (from 9 to 33 points), skinfold (calf) (from 9 to 33 points), excess weight (from 9 to 34 points), obesity (from 9 to 33 points), and abdominal obesity (from 18 to 32 points). Conversely, a second area showed that high PPLI was inversely related to BMI (>34 points), BMI *z* score (>34 points), WC (≥33 points), WHtR (>34 points), skinfold (triceps) (≥33 points), skinfold (calf) (>33 points), excess weight (≥35 points), obesity (≥34 points), and abdominal obesity (>35 points). Lastly, estimated marginal means and predictive probabilities and their 95% CIs of each obesity-related indicator according to the S-PPLI score derived from GAMs are found in Supplementary Figure S1.

Table 2 displays the estimated marginal means and predictive probabilities (with their 95% CIs) of each obesity-related indicator academic performance in relation to PPL status. Adolescents with lower PPL showed the highest estimated marginal means of BMI, BMI *z* score, WC, WHtR, and skinfold (triceps and calf) and predictive probabilities of having excess weight, obesity, and abdominal obesity, while their counterparts with high PPL had the lowest. In addition, significant differences were observed for all the obesity-related indications between adolescents with low PPL and those with medium PPL (*p*-adjusted < 0.05 for all indicators), as well as with those with high PPL (*p*-adjusted < 0.05 for all indicators). Moreover, these significant differences were also shown for most indicators between adolescents with medium PPL and those with high PPL (except for obesity).

**Table 2:** Estimated marginal means and predictive probabilities derived from generalized linear models of each obesity-related indicator among adolescents according to perceived physical literacy status.

Obesity-related indicator	Low PPL^§^ (9 to 31 points) (*n* = 292; 34.6%)	Medium PPL^§^ (32 to 36 points) (*n* = 240; 28.4%)	High PPL^§^ (37 to 45 points) (*n* = 313; 37.0%)
BMI (kg/m^2^)	24.1 (23.4 to 24.8)	23.0 (22.4 to 23.7)^a^	22.2 (21.5 to 22.9)^a,b^
BMI (*z* score)^†^	0.29 (0.09 to 0.50)	0.00 (−0.19 to 0.19)^a^	−0.27 (−0.47 to −0.06)^a,b^
WC (cm)	78.1 (76.7 to 79.5)	75.9 (74.6 to 77.2)^a^	73.7 (72.3 to 75.2)^a,b^
WHtR ([WC (cm)/height (cm)])	0.475 (0.466 to 0.483)	0.462 (0.454 to 0.470)^a^	0.446 (0.437 to 0.455)^a,b^
Skinfold triceps (mm)	16.5 (15.5 to 17.5)	15.2 (14.3 to 16.1)^a^	13.2 (12.2 to 14.2)^a,b^
Skinfold calf (mm)	17.9 (16.7 to 19.0)	16.3 (15.3 to 17.4)^a^	14.4 (13.3 to 15.6)^a,b^
Excess weight (%)^†^	37.2 (30.0 to 45.1)	26.7 (21.0 to 33.2)^a^	19.2 (14.1 to 25.6)^a,b^
Obesity (%)^†^	16.5 (11.1 to 23.8)	9.2 (5.8 to 14.2)^a^	7.7 (4.5 to 12.8)^a^
Abdominal obesity (%)^‡^	38.6 (30.9 to 46.9)	24.6 (19.0 to 31.3)^a^	14.2 (9.8 to 20.3)^a,b^

The data are expressed as estimated marginal means or predictive probabilities and bias-corrected and accelerated bootstrapped 95% confidence intervals. Adjusted for sex, age, socioeconomic status, adherence to the Mediterranean diet, energy intake, overall sleep duration, physical activity, and sedentary behavior. False discovery rate *p*-value method proposed by Benjamini and Hochberg applied (35)BMI, body mass index; PPL, perceived physical literacy; WC, waist circumference; WHtR, waist-to-height ratio.^§^According to the Spanish Perceived Physical Literacy Instrument (24).^†^According to the World Health Organization (26).^‡^Using a cut-off point of waist-to-height ratio ≥ 0.5 (27).^a^Statistically significant difference compared to adolescents with low PPL (p < 0.05).^b^Statistically significant difference compared to adolescents with medium PPL (*p* < 0.05).

## 4 Discussion

Overall, our findings showed that higher PPL is related to lower BMI, BMI z score, WC, WHtR, and skinfold (triceps and calf), as well as with a lower probability of having excess weight, obesity, and abdominal obesity. Although there is limited empirical evidence linking PPL and health outcomes, the literature supports a relationship between the greater physical domain of physical literacy and more desirable health outcomes. A review by Cornish et al. (36) pointed out that physical literacy is related to lower body weight, BMI, waist circumference, physical activity, sedentary behavior, and cardiorespiratory fitness. Likewise, a meta-analysis by Carl et al. (37) revealed that physical literacy interventions increase physical competence, physical activity behavior, knowledge and understanding in relation to physical activity, overall physical literacy, motivation and confidence (despite meaningful heterogeneity). More specifically, among adolescents, these results agree with those obtained by Caldwell et al. (21) and Nezondet et al. (22). The usefulness of applying a physical literacy perspective to promote the practice of physical activity among young people has been highlighted, which may be particularly relevant for young people with obesity (38). Furthermore, a study by Nezondet et al. (22) found that a physical literacy-based intervention reduced BMI z score and body fat percentage among adolescents with excess weight. Although only a few studies have examined the relationship between physical literacy and obesity-related indicators among adolescents, there are some possible mechanisms that could justify these findings.

On the one hand, one possible explanation could lie in the association between physical literacy and physical fitness. Physical literacy is a multifaceted concept that includes several domains (e.g., physical competence) (36). In this sense, the Canadian Assessment of Physical Literacy – 2nd edition (CAPL-2) includes a test to measure physical fitness (i.e., cardiorespiratory fitness, muscular strength, speed-agility) as a domain of physical competence (39). Specifically, among youths, Pastor-Cisneros et al. (40) observed that physical literacy was associated with higher self-perceived physical fitness among Spanish youths aged 8 to 12 years. Similarly, Gilic et al. (41) reported that physical literacy was related to higher physical fitness in Croatian adolescents. It must be considered that one of the most powerful markers of health is physical fitness (42). In this sense, several studies have analyzed the relationship between physical fitness and certain cardiometabolic risk factors among youth (e.g., blood pressure, insulin resistance) (43, 44) because all of them have been shown to track from childhood into adulthood (45). Although the S-PPLI measures physical literacy in a self-perceived manner, it is possible that adolescents with higher scores on that tool also possess higher levels of physical fitness, which could explain the lower levels in the obesity-related indicators examined.

On the other hand, higher physical literacy may lead to greater participation in physical activities (46). Physically literate adolescents have a better understanding of how to engage in different physical activities and sports (47), which could motivate them to participate in a variety of physical activities. Although the etiology of obesity is complex (48), this fact can help maintain a healthy weight. In this sense, physical activity has also been associated with lower obesity-related indicators among children and adolescents (49, 50). Physical activity is an effective way to prevent weight gain and maintain a healthy energy balance (51), as it increases people’s total energy expenditure, which could help them stay in energy balance or even lose weight, as long as they do not eat more to compensate for the extra calories they burn (52). This increase in energy expenditure could, at least partially, explain the results obtained.

Additionally, physical literacy is frequently the literacy that other literatures must pass through (53). Through physical activity, individuals can develop not only their own physical literacy but also a global or holistic literacy that aids in navigating, connecting with, and understanding themselves, others, and the environment in which they live (53). Supporting this idea, health literacy synergistically complements physical literacy to facilitate the adoption of healthy lifestyles (54). Adolescents with health literacy could develop robust personal health habits (e.g., adequate diet, recommended physical activity) and refrain from unhealthy behaviors (e.g., drug abuse) (55). Likewise, several studies have reported that health literacy is associated with more desirable health outcomes among adolescents (56, 57). Moreover, PPL also involves knowledge and understanding about other healthy habits (e.g., the importance of balanced nutrition). Food literacy indicates proficiency in food-related knowledge and skills (58) and may play a role in shaping adolescents’ dietary intake (59). Furthermore, some studies have indicated that food literacy is inversely related to excess weight among adolescents (60, 61). Adolescents with higher PPL may be better able to make informed decisions about their health and well-being and be aware of the risks associated with unhealthy lifestyles (e.g., inadequate diet) (62), which could help prevent obesity.

Another possible reason could lie in the relationship between physical education and PL. Physical literacy is the cornerstone of physical education, which is most readily attained when students have access to a variety of opportunities that are appropriate for their age and stage (63). In this sense, physical education attendance has been related to a healthier lifestyle (64–66). For instance, Lirola et al. (66) showed that physical education classes can positively influence the adoption of healthy eating habits. In addition, García-Hermoso et al. (64) observed that physical education attendance [which is closely related to physical literacy (67)] was linked with meeting all three 24-h movement recommendations (i.e., physical activity, screen time, and sleep duration) in adolescents, and this association was maintained in adulthood. Supporting this idea, a meta-analysis by López-Gil et al. (7) has revealed that meeting all these 24-h movement recommendations has been related to lower obesity-related outcomes (i.e., excess weight, obesity, BMI, BMI z score, waist circumference, and body fat). Given the propensity of healthy behaviors to cluster together (e.g., diet, physical activity) (68–71), it is possible that a greater PPL led to a healthier lifestyle, which could favor an optimal body weight and body composition in adolescents.

The present study had some limitations that must be declared. First, due to the cross-sectional design of this study, a direct causal link based on the results cannot be established. Similarly, we are also unable to verify the direction of the association. While further research employing diverse methodologies, such as experimental approaches, is needed to examine whether a higher PPL is linked with more desirable obesity-related outcomes among adolescents, as well as to uncover the underlying mechanisms. It must be considered that existing data suggest a reciprocal connection between obesity-related outcomes and PPL [i.e., young people with higher levels of obesity-related parameters have lower levels of physical literacy (72–74)]. Likewise, employing questionnaires for collecting data about PPL (and other covariates) may introduce social desirability or recall bias, which may influence the results obtained. Conversely, a strength of this study is that it examines a little studied association in adolescents, which increases the scientific knowledge in this field. Similarly, these analyses include a wide range of objective obesity-related indicators (i.e., BMI, BMI *z* score, waist circumference, WHtR, skinfolds, excess weight, obesity, and abdominal obesity), providing a deeper understanding of this association. Moreover, our analyses were adjusted for sociodemographic (i.e., age, sex, socioeconomic status) and lifestyle variables (i.e., energy intake, adherence to the Mediterranean diet, sleep duration, physical activity, sedentary behavior), which confers more robustness to these results.

## 5 Conclusion

Physical literacy could play a crucial role in maintaining more desirable obesity-related outcomes in adolescents. Adolescents with high PPL showed lower obesity-related indicators (i.e., BMI, BMI *z* score, WC, WHtR, skinfolds), as well as a lower probability of having excess weight, obesity, and abdominal obesity. These findings support the idea of promoting physical literacy as a means of improving young people’s health (36) and could be used in the development of interventions aimed at preventing obesity in adolescents. These interventions could include structured physical education programs that not only focus on increasing physical activity levels but also aim to improve physical literacy in a holistic manner, as a strategic approach to addressing adolescent obesity.

## Data availability statement

The raw data supporting the conclusions of this article will be made available by the authors, without undue reservation.

## Ethics statement

The studies involving humans were approved by the Bioethics Committee at the University of Murcia (Approval ID: 2218/2018), the Ethics Committee of the Albacete University Hospital Complex, and the Albacete Integrated Care Management (Approval ID: 2021-85). The research was conducted in accordance with the principles outlined in the Helsinki Declaration. The studies were conducted in accordance with the local legislation and institutional requirements. Written informed consent for participation in this study was provided by the participants’ legal guardians/next of kin. Written informed consent was obtained from the individual(s), and minor(s)’ legal guardian/next of kin, for the publication of any potentially identifiable images or data included in this article.

## Author contributions

GD-M: Writing – original draft. PT-L: Writing – review & editing. JL-G: Conceptualization, Data curation, Formal analysis, Investigation, Methodology, Software, Supervision, Writing – review & editing.
