# Supplementary figures and images for: Relationship between perceived physical literacy and obesity-related outcomes in adolescents: the EHDLA study
Source: Front Public Health. 2024 Apr 16;12:1321361. doi: 10.3389/fpubh.2024.1321361 (PMC11062133; doi:10.3389/fpubh.2024.1321361)

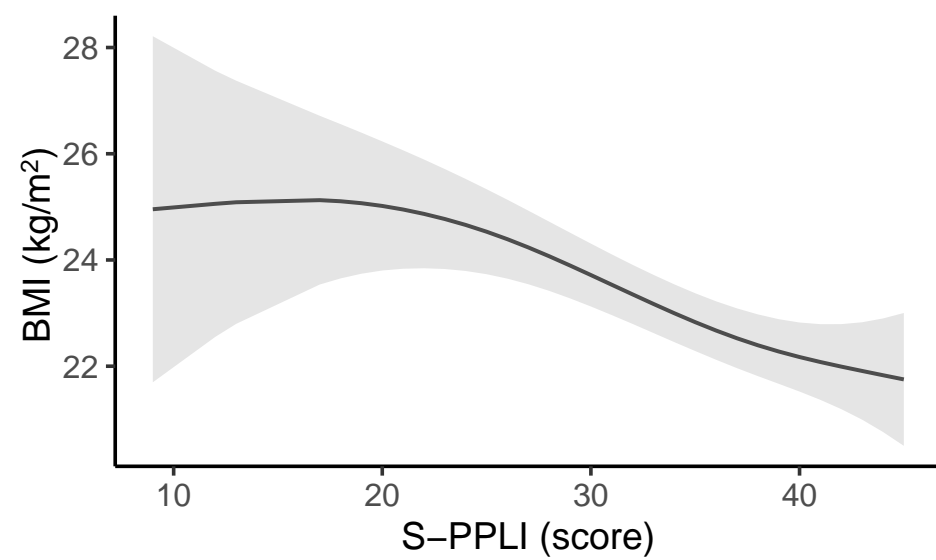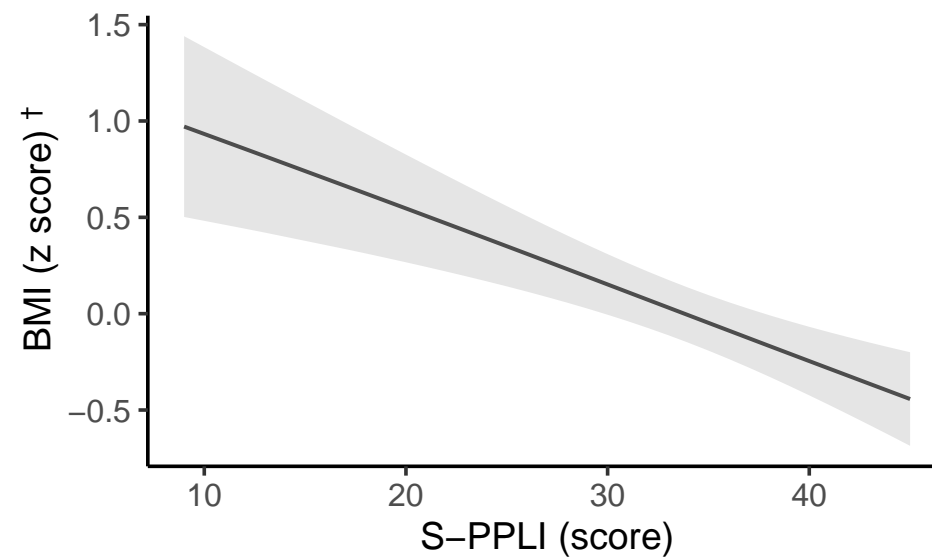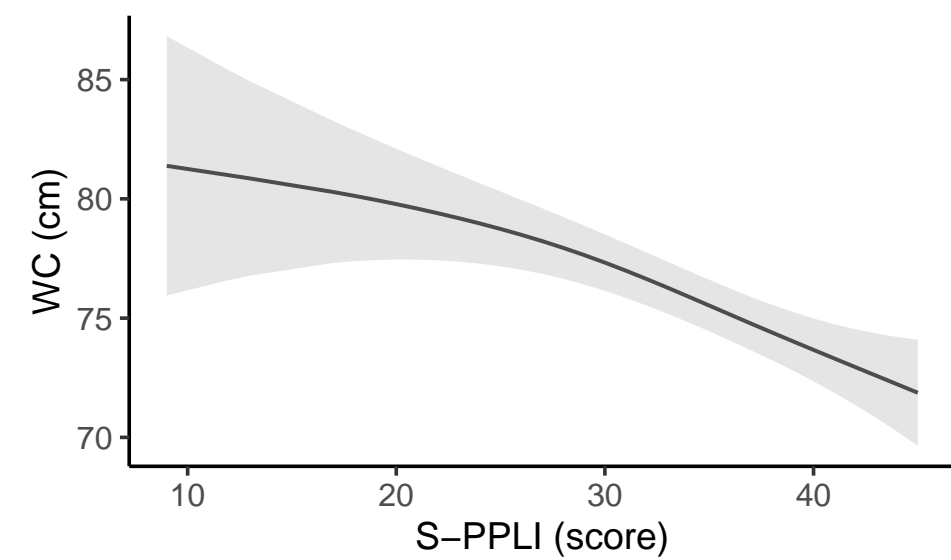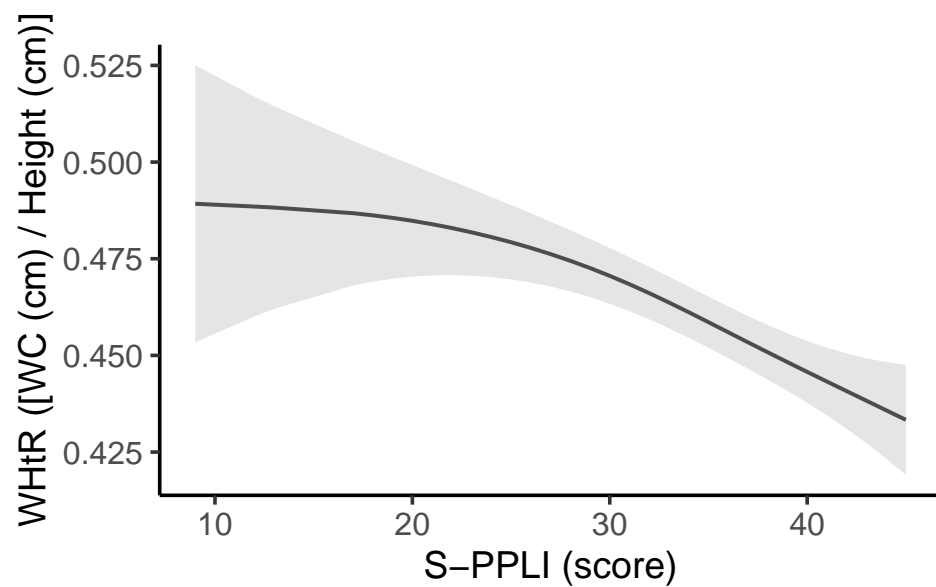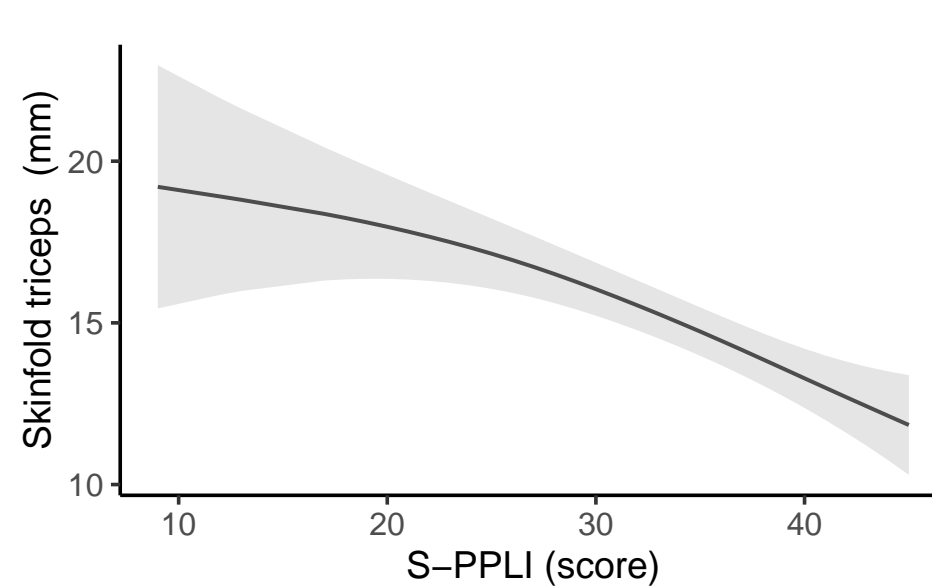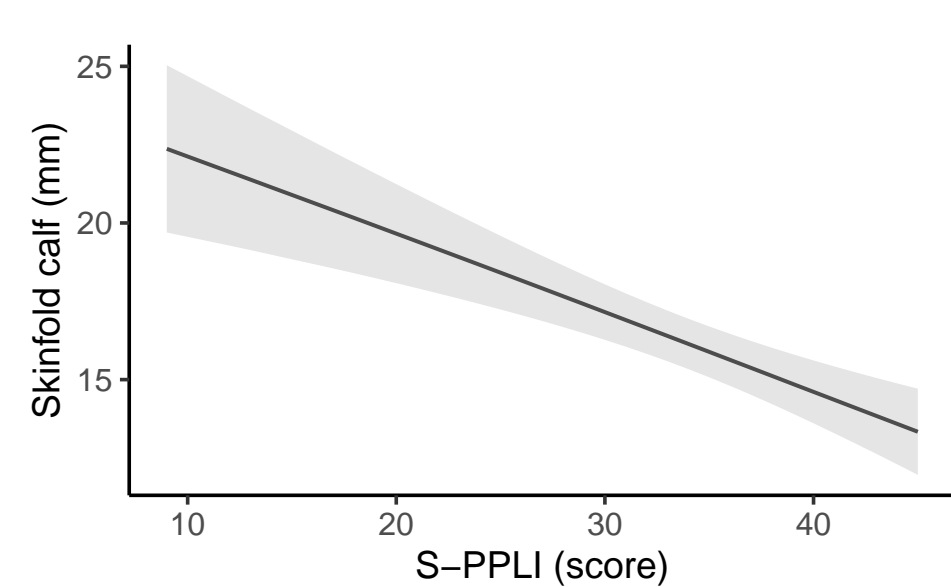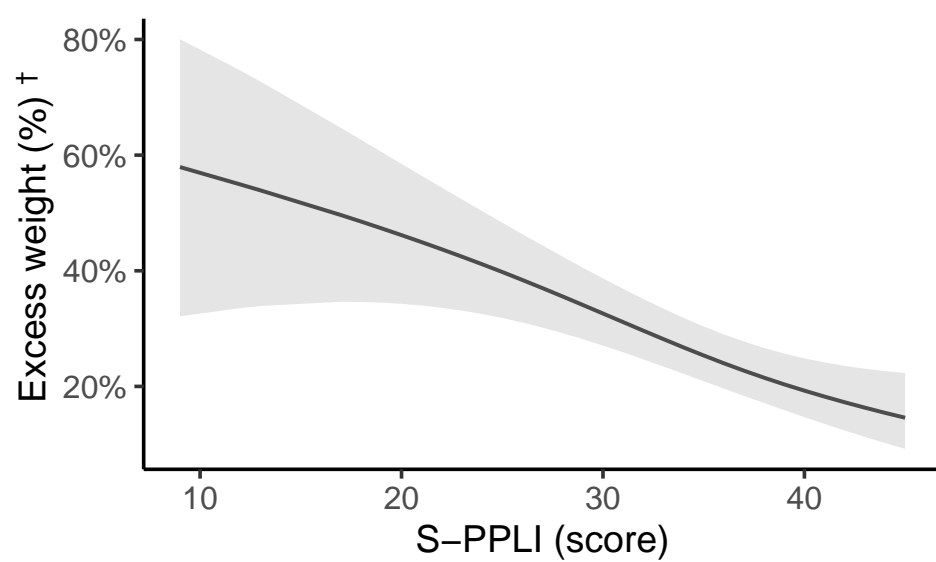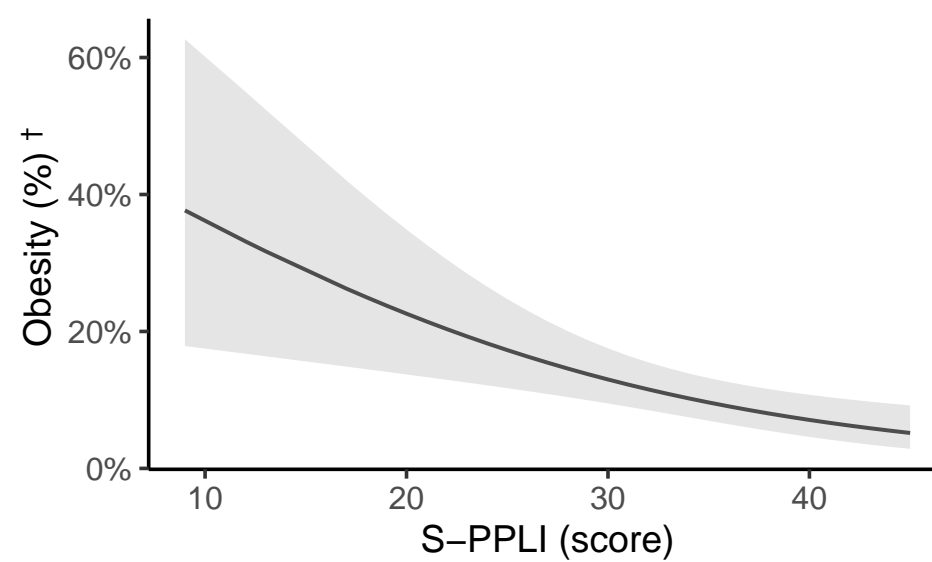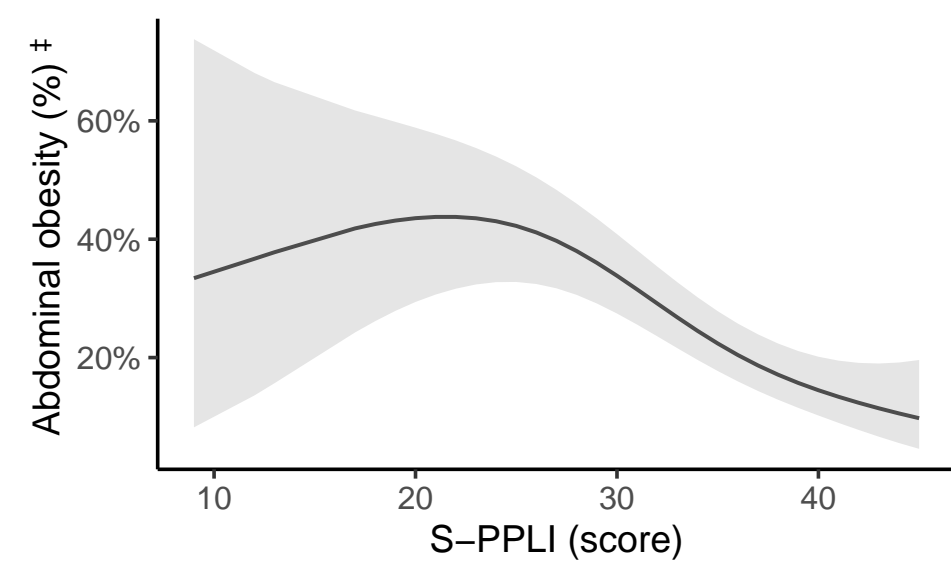

Supplement: Supplementary file 1 [file Data_Sheet_1.PDF]
